# BLM promotes the activation of Fanconi Anemia signaling pathway

**DOI:** 10.18632/oncotarget.8707

**Published:** 2016-04-12

**Authors:** Jayabal Panneerselvam, Hong Wang, Jun Zhang, Raymond Che, Herbert Yu, Peiwen Fei

**Affiliations:** ^1^ University of Hawaii Cancer Center, University of Hawaii, Honolulu, HI, USA; ^2^ Department of Laboratory Medicine and Pathology, Mayo Clinic, Rochester, MN, USA; ^3^ Current address: Sun Yat-Sen University, Guangzhou, China

**Keywords:** Fanconi Anemia, BLM, FANCD2 monoubiquitination, DNA damage, tumorigenesis

## Abstract

Mutations in the human RecQ helicase, BLM, causes Bloom Syndrome, which is a rare autosomal recessive disorder and characterized by genomic instability and an increased risk of cancer. Fanconi Anemia (FA), resulting from mutations in any of the 19 known FA genes and those yet to be known, is also characterized by chromosomal instability and a high incidence of cancer. BLM helicase and FA proteins, therefore, may work in a common tumor-suppressor signaling pathway. To date, it remains largely unclear as to how BLM and FA proteins work concurrently in the maintenance of genome stability. Here we report that BLM is involved in the early activation of FA group D2 protein (FANCD2). We found that FANCD2 activation is substantially delayed and attenuated in crosslinking agent-treated cells harboring deficient Blm compared to similarly treated control cells with sufficient BLM. We also identified that the domain VI of BLM plays an essential role in promoting FANCD2 activation in cells treated with DNA crosslinking agents, especially ultraviolet B. The similar biological effects performed by ΔVI-BLM and inactivated FANCD2 further confirm the relationship between BLM and FANCD2. Mutations within the domain VI of BLM detected in human cancer samples demonstrate the functional importance of this domain, suggesting human tumorigenicity resulting from mtBLM may be at least partly attributed to mitigated FANCD2 activation. Collectively, our data show a previously unknown regulatory liaison in advancing our understanding of how the cancer susceptibility gene products act in concert to maintain genome stability.

## INTRODUCTION

The effective repair of DNA damage, caused by exogenous agents or during DNA replication, confers protection from neoplastic transformation. Several genetic disorders that perturb the repair of DNA damage result in an increased predisposition to cancer [1–5]. One such disorder is a rare multigenic syndrome known as Fanconi Anemia (FA), which is characterized by subfertility, congenital abnormalities, progressive bone marrow failure, and an increased risk of hematological and non-hematological malignancies [6–10]. To date, 19 FA genes have been identified, and their encoding proteins can act through a common signaling pathway, namely the FA or FA-BRCA pathway [11–16]. Following various types of DNA damage and during the S- phase of the cell cycle, a nuclear ‘core complex’ containing eight FA proteins (FANCA, -B, -C, -E, -F, -G, -L and -M) is required for the monoubiquitination of FANCD2 and FANCI, which are recruited to chromatin [17, 18]. Chromatin bound FANCD2 and FANCI colocolize into nuclear foci that represent the center of ongoing DNA repair [19]. Recently, much focus has been placed on ubiquitination as an important modification in determining how the monoubiquitination of FANCD2 (or the activation of the FA pathway) is achieved. However, this activation has yet to be fully elucidated.

Bloom Syndrome (BS) [20], similar to FA, is an autosomal recessive disease, which results essentially from a deficiency in DNA-repair leading to chromosomal instability, aging and a high susceptibility to cancer [21]. The gene mutated in BS, namely BLM, encodes a 159kDa RecQ family DNA helicase [22, 23]. The BLM protein is a 3′–5′ DNA helicase that processes a broad range of structurally diverse DNA substrates [24–26]. These substrates include DNA structures that arise during homologous recombination, such as D-loops and Holliday junctions [27]. RecQ proteins appear to activate recombination, thus enhancing global genome stability, and are involved in the response to a variety of DNA adducts [28, 29]. In general, DNA helicases function in many aspects of DNA metabolism including DNA repair, replication, recombination, transcription, and RNA processing, although the exact function of the BLM helicase in these processes still remain uncharacterized. Additionally, the biochemical basis for the increased sun sensitivity of BS patients is unknown but suggests there exists an abnormal response to crosslinking DNA damage. In cells treated with DNA crosslinking agents, the Blm complex associates with the FA core complex to form a 1.5–2MDa super complex termed BRAFT [3]. This suggests that the overlapping phenotypes of FA and BS may be due to a failure in BRAFT assembly and other mechanisms, which have yet to be defined. This study documents that BLM is important in maintaining the activation of the FA pathway at a proper magnitude, and its motif VI is crucial for this activation. Furthermore, naturally occurring mutations in the motif VI of BLM were found to be present in human tumors vividly show the functional significance of our findings.

## RESULTS

### FANCD2 activation is delayed in BS cells

**Figure 1:**
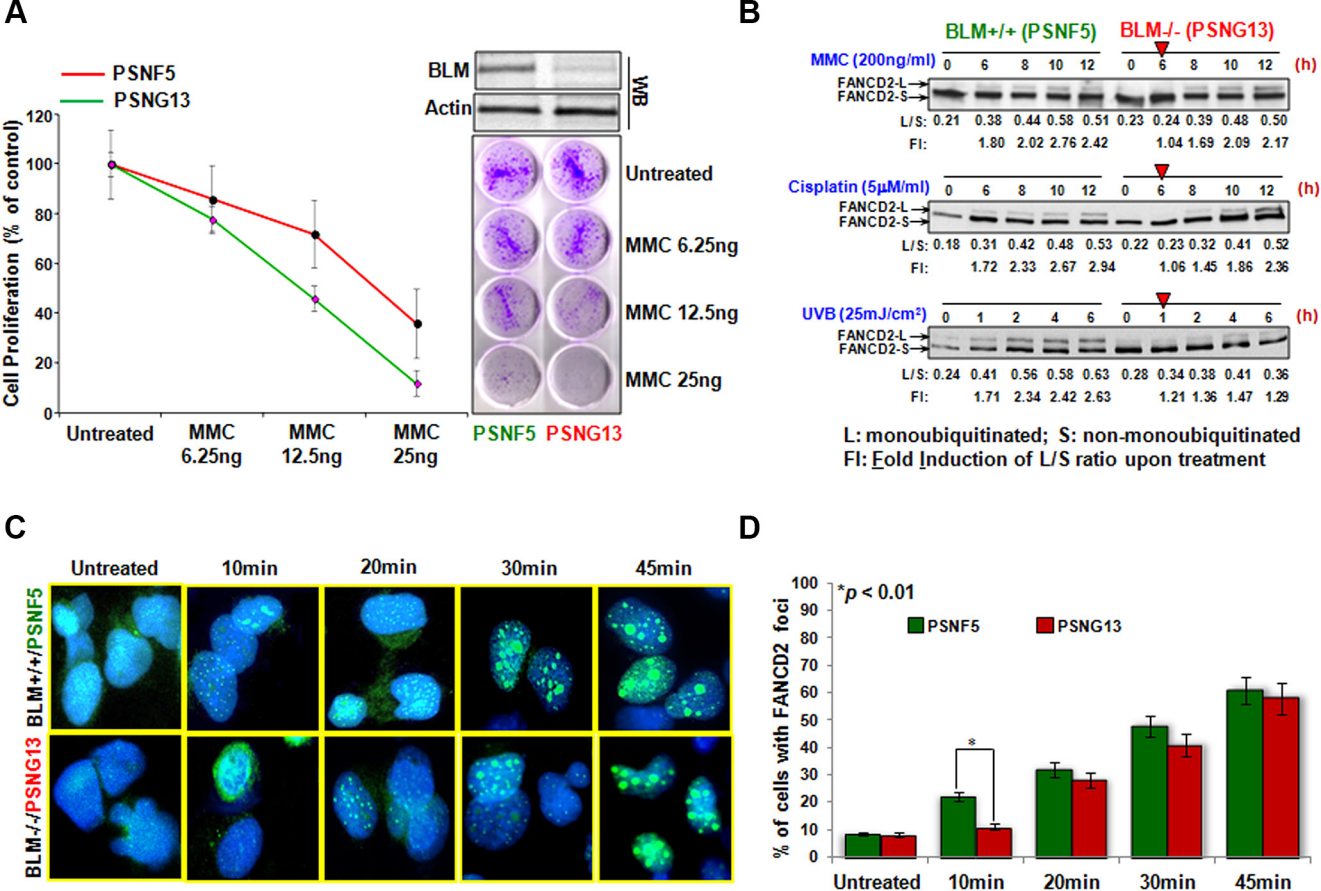
FANCD2 activation is sharply mitigated in BS cells (**A**) BS cells are sensitive to MMC treatment. Both BLM proficient (PSNF5) and deficient (PSNG13) cells were treated with the indicated concentration of MMC. PSNG13 (BLM deficient) cells were more sensitive to the treatment of MMC than BLM proficient cells. (**B**) The magnitude of FANCD2 activation is reduced in BS cells treated with MMC, Cisplatin and UVB. We treated both the BLM proficient (PSNF5) and deficient (PSNG13) cells with MMC (200 ng/ml; top panel), Cisplatin (5 μM/ml; middle panel) and UVB (25 mJ/cm^2^; bottom panel) respectively, and harvested cells at the indicated time points. Whole cell extracts were used to conduct Western blotting with anti-FANCD2 antibodies. As red arrowheads indicated, BLM deficient cells showed a delayed activation of FANCD2 as compared to BLM proficient cells. The band density was determined by Image J software, and the ratio of FANCD2-L (monoubiquitinated FANCD2 isoform) to FANCD2-S (un-monoubiquitinated form) was calculated (L/S). Fold induction (FI) is the L/S ratio of the treated over the untreated.(**C**) FANCD2 foci are reduced in BS cells. The same batch of UVC-treated cells (Supplementary Figure S1A) were also prepared for the immunofluorescent study of FANCD2 monoubiquitination/activation. BLM deficient cells carry a lower number of FANCD foci compared to BLM proficient cells (Foci represent the monoubiquitinated FANCD2) (images were taken at a magnification of 400X). (**D**) Quantification of FANCD2 focus formation. The number of nuclei positive for FANCD2 foci was measured in 120 cells for each time point. The graph shows the mean and SD of the percentage of cells with > 10 FANCD2 foci. Statistical significance was calculated using the Pearson's Chi-square test. Data are the means of 3 independent determinations.

A functional link between BLM and the FA pathway has now been suggested for a decade. However, there is a lack of evidence to demonstrate a direct functional connection between BLM and the FA proteins. Thus we utilized a pair of human fibroblast cell lines, PSNG13 derived from a BS patient and its derivative PSNF5, complemented with functional BLM [30] to examine how BLM influences the FA signaling pathway. As FA cells are featured with the hypersensitivity to DNA crosslinking agents, we examined how BS cells (PSNG13 and PSNF5) responded to the same agent(s). We found that Blm null cells (PSNG13) were more sensitive to the treatment of mitomycin C (MMC) than their corresponding control cells (Figure 1A), supporting the functional link between BLM and FA signaling. The subsequent time course studies demonstrated that FANCD2 monoubiquitination is sharply reduced in Blm deficient cells upon the treatment of ultraviolet (UV) B/C, MMC or Cisplatin (Figure 1B and Supplementary Figure S1A). FANCD2 focus formation is an alternative approach to represent FANCD2 activation. We thus conducted an immunofluorescent (IF) study of FANCD2 in the same batch of cells used for Western blotting (Supplementary Figure S1A), and found that the FANCD2 foci were dramatically reduced in cells deficient in Blm (Figure 1C and 1D). Together, for the first time these results demonstrate that BLM plays a role in promoting FANCD2 activation in response to DNA damage.

### The activation of FANCD2 is also delayed in human cancer cells expressing a low level of BLM protein

**Figure 2:**
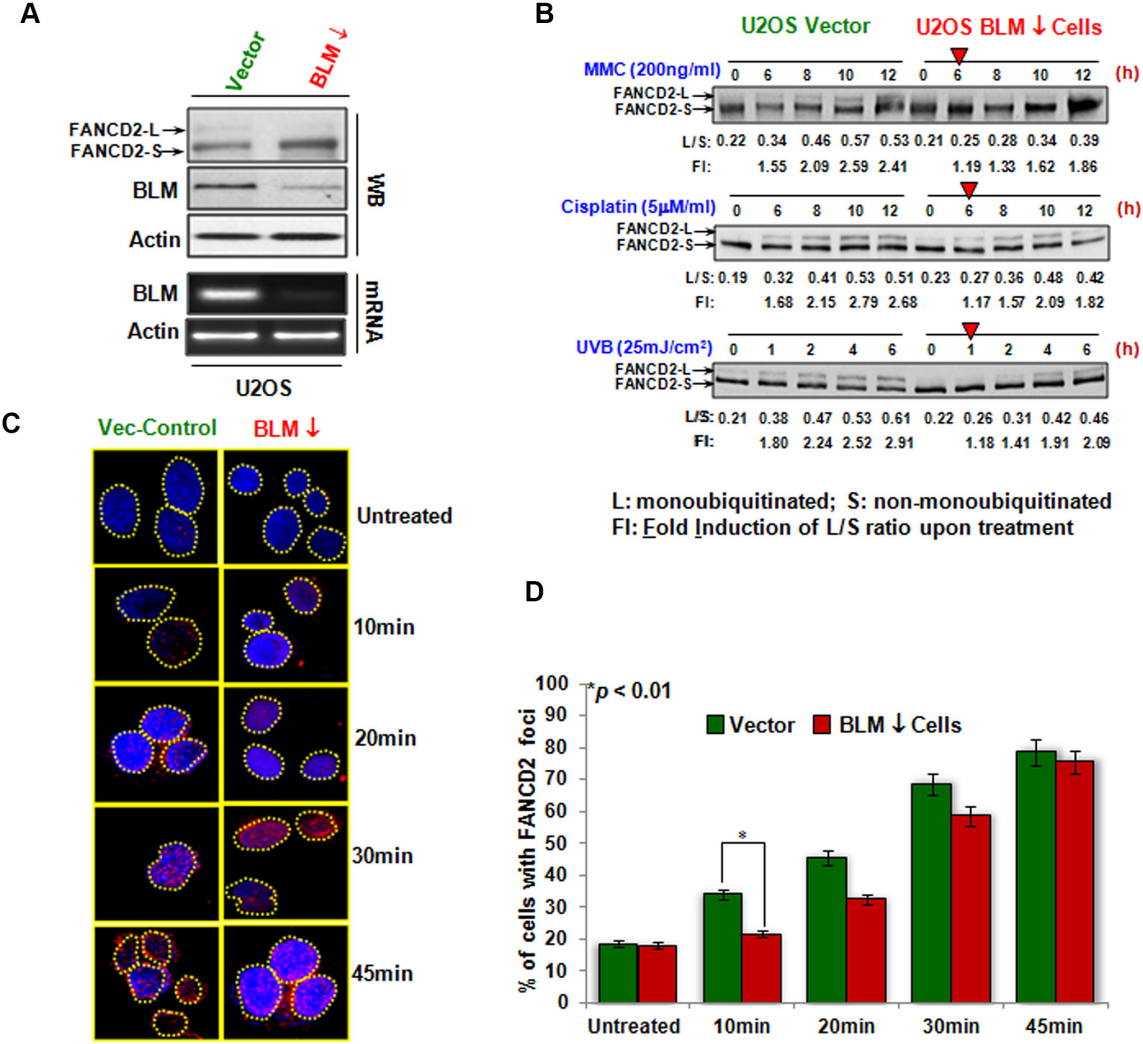
The regulation of FANCD2 activation by BLM in non-BS human cancer cells (**A**) U2OS cells carrying shRNA targeting Blm or empty vector were established. The levels of both BLM mRNA and protein were downregulated compared to control cells. The corresponding level of monoubiquitinated FANCD2 is compromised in BLM-downregulated cells. U2OS stable cells isogenic to the basal level of monoubiquitinated FANCD2 expression were established via silencing FANCL. (**B**) FANCD2 activation is compromised in BLM-deficient U2OS cells. As red arrowheads indicated, BLM-downregulated cells showed a clear delayed activation of FANCD2 as compared to their corresponding control cells. L/S ratio is the level of S (un-monoubiquitinated) over L (monoubiquitinated) forms of FANCD2. Fold induction (FI) is the L/S ratio derived from the treated over the one from untreated cells. (**C**) FANCD2 foci in BLM-silenced U2OS cells are decreased. The same batch of U2OS stable cells, used for Western blotting in A, was also prepared for the immunofluorescent study of monoubiquitinated FANCD2. Cells were treated with UVC (50 J/m^2^) as described in the Materials and Methods. Again, BLM-downregulated cells carry a lower density of FANCD foci compared to vector cells (Foci represent the monoubiquitinated FANCD2). (**D**) Quantification of FANCD2 focus formation. The number of nuclei positive for FANCD2 foci was measured in 120 cells for each time point. The graph shows the mean and SD of the percentage of cells with > 10 FANCD2 foci. Statistical significance was calculated using the Pearson's Chi-square test. Data are the means of 3 independent scores.

BLM helicase is well acknowledged at many aspects of DNA metabolism including DNA replication, repair, recombination, and transcription. To this point, we wanted to see if this novel regulatory link was restricted to BS cells. We generated a set of stable cell pairs isogenic to the level of Blm expression using human osteosarcoma U2OS cells. This was achieved via lentivirus-mediated delivery of shRNA targeting Blm mRNA (Figure 2A), noting that the basal level of monoubiquitinated FANCD2 was undetectable in BLM-downregulated cells. Using these U2OS derivative cells expressing either normal or a downregulated level of BLM protein, we performed similar experiments using different genotoxic agents such as UVB/C, MMC, and Cisplatin (Figure 2B and Supplementary Figure S1B). We found that U2OS cells carrying a reduced level of BLM expression exhibited a delayed activation of FANCD2 similar to what was observed in BS cells (Figure 1B). We also conducted a similar IF study on FANCD2 and found that the FANCD2 foci were dramatically reduced in BLM-downregulated U2OS cells at the earlier time points compared to the corresponding control cells (Figure 2C and 2D). Furthermore, the results obtained from the PA-1 derivative cells (Supplementary Figure S2A) confirmed these findings (Supplementary Figure S2B–S2D). Therefore, the enhancement of FANCD2 activation by BLM in response to genotoxic stresses was not restricted to BS cells, unveiling a common link that coordinates genomic surveillance.

### Domain VI of BLM protein contributes to the early activation/monoubiquitination of FANCD2

**Figure 3:**
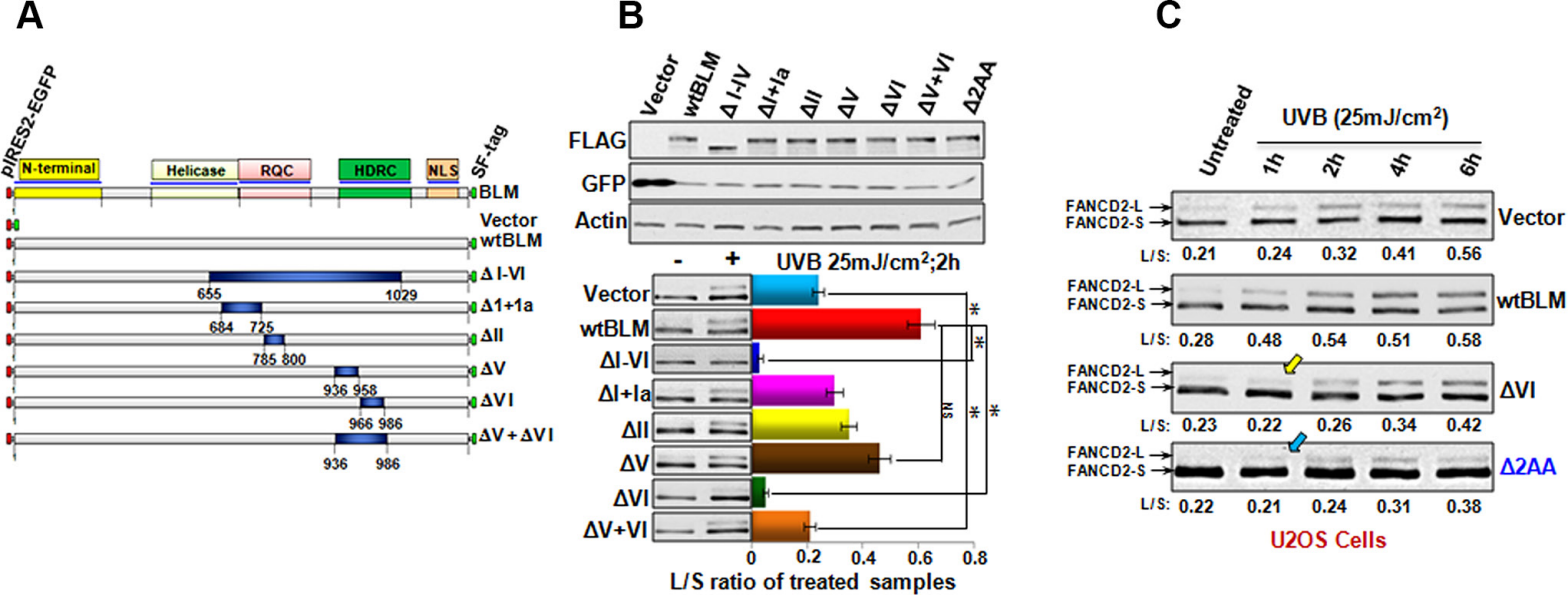
Motif VI of BLM contributes to the early FANCD2 activation (**A**) Schematic diagram illustrates the full-length BLM and various deletion mutants of BLM. The deleted part is shown in dark blue, and the remaining portion is shown in light gray. (**B**) B-top; Transfection efficiency of various versions of BLM plasmids in U2OS cells is equal, and examined with GFP-antibody [the various versions of BLM cDNAs were cloned in a polycistronic expression plasmid (PIRES2-EGFP). BLMs and GFP share the same mRNA but both are translated into proteins separately]. B-bottom; U2OS cells were transfected with plasmids containing various versions of BLM cDNAs encoding mtBLM proteins with Δ 6 motifs (a complete ΔI-VI), Δ(I+Ia), ΔII, ΔV, ΔVI, Δ(V+VI) and Δ2AA at Y974Q975 of BLM respectively, or empty vector as the control. All plasmids used were PIRES2-EGFP-derivatives. Cells were treated with UVB at 25 mJ/cm^2^ and harvested at the 2 h time point. U2OS cells transfected with ΔVI-BLM, or a complete deletion (ΔI-VI) show a compromised level of FANCD2 activation/monoubiquitination (green or blue bar respectively), as compared to the cells carrying wtBlm (red bar). The asterisks denote *p < 0.01. (**C**) U2OS cells transfected with the vector, wtBLM, ΔVI BLM and, ΔY974Q975 BLM were treated with UVB 25 mJ/cm^2^ and harvested at the indicated time points. Cells carrying either ΔVI-BLM or ΔY974Q975-BLM (Δ2AA) showed a compromised early activation of FANCD2 as well as a reduced intensity of the activation in comparison with the cells transfected with wtBLM.

The core functional domain of the BLM protein is the RecQ helicase domain. This domain is composed of seven conserved motifs designated I, Ia, II-VI. The structural model for the BLM helicase demonstrates that Ia, III and V directly bind to the ssDNA of the helicase substrate, while I, II, III, IV and VI form the binding pocket for ATP binding and hydrolysis [31]. To gain insight into how BLM promotes FANCD2 early activation, we wanted to see which specific motif of BLM would be paramount. We constructed a set of BLM-derived plasmids containing Δ(I-VI), Δ(I+Ia), ΔII, ΔV, ΔVI and Δ(VI+VI), respectively (Figure 3A). These BLM expression constructs were transiently transfected into U2OS cells (Figure 3B top). The transfected U2OS cells were treated with UVB (25 mJ/cm^2^) to screen which motif of BLM influenced FANCD2 monoubiquitination. Cells carrying ΔVI-BLM were observed to have an effect on FANCD2 monoubiquitination/activation (Figure 3B bottom). Next, we investigated whether there was a specific time point for the motif VI to influence the activation of FANCD2. We transfected the empty vector, motif VI deleted-Blm cDNA, and wtBlm cDNA into U2OS cells respectively, which were treated with UVB (25 mJ/cm^2^). As shown in Figure 3C-marked with a yellow arrowhead, cells harboring mtBLM (deleted VI), delayed the activation of FANCD2 compared to cells expressing wtBLM. This finding revealed a previously unknown role that the motif VI of BLM plays in the enhancement of FANCD2 activation.

### A naturally occurring deletion mutation in domain VI of bloom can mitigate BLM regulation of FANCD2 early activation

To explore the functional significance of this novel signaling link, we searched various available databases for BLM mutations occurring in BS patients. We found there were deletion and/or point mutations in the Y974Q975 region within the motif VI of BLM protein that were documented in human cancers [32, 33]. Furthermore, we tested whether this naturally-occurring mutation region is able to affect the activation of FANCD2. Following this, we found that U2OS cells transfected with Blm cDNA encoding the two-AA deletion mutation showed a compromised activation of FANCD2 in comparison with the cells transfected with wtBlm cDNA (Figure 3C-marked with a blue arrowhead). This result not only indicates functional importance of BLM enhancement in FANCD2 monoubiquitination/activation, but also advances our understanding of the molecular basis underlying BS, at least partly attributed to a compromised FA signaling pathway.

### Cells carrying a compromised FA signaling or expressing a low level of BLM show the similar biological effects

**Figure 4:**
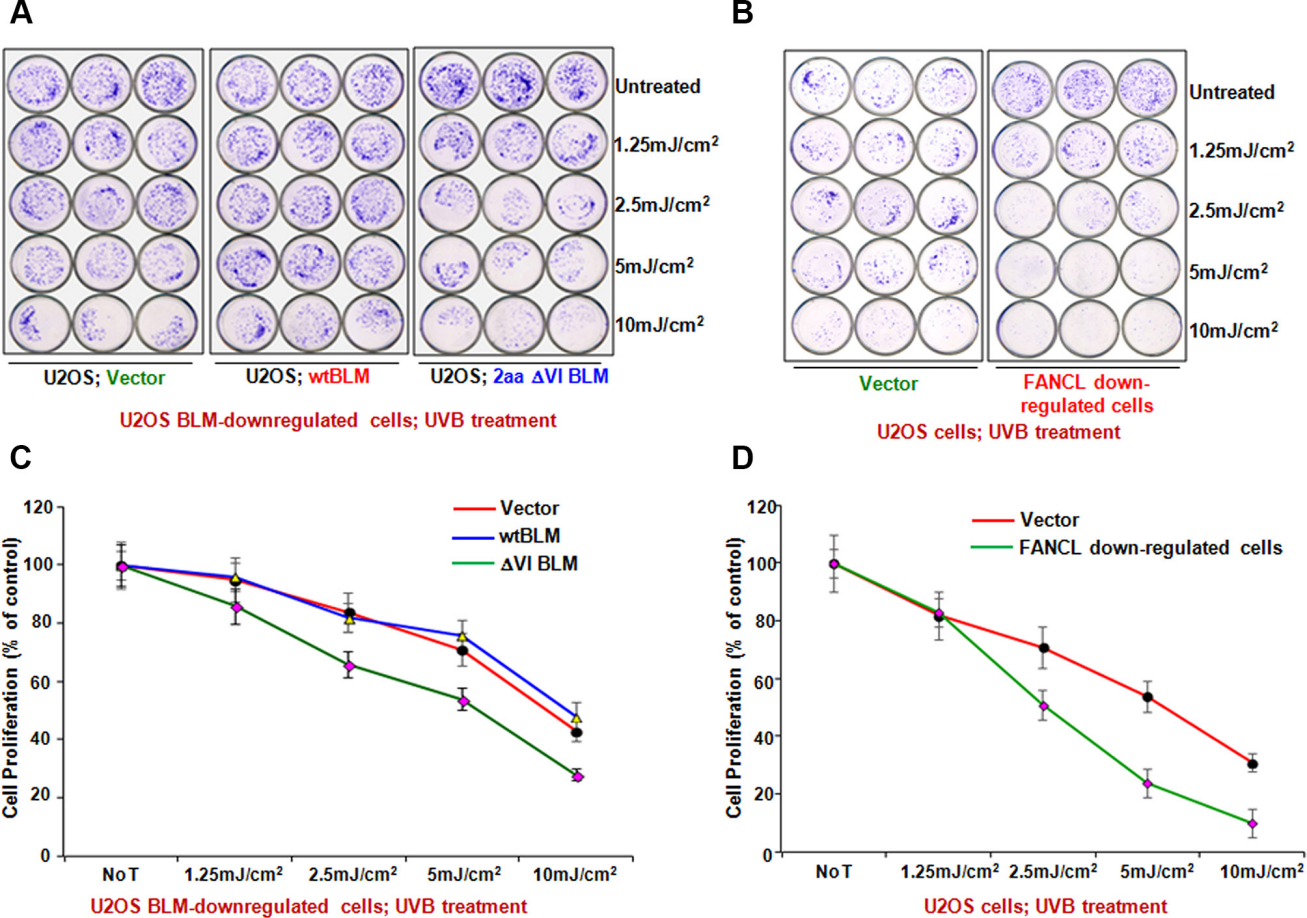
Biological significance of BLM Regulation through FANCD2 Activation (**A**) BLM-downregulated or (**B**) FANCL-downregulated U2OS cells were more sensitive to UVB treatment than their corresponding control cells. This is similar to what is seen in these cells treated with MMC (Supplementary Figure S3A and S3B). Representative results of at least three independent experiments assessed by crystal violet staining are shown and the survival curves plotted upon the quantification of cell proliferation are shown in (**C** and **D**) respectively (Means ± SD of 3 independent experiments). The asterisks denote *P < 0.05.

To further confirm this previously unknown signaling link between BLM and FANCD2 activation, we anticipated that cells harboring the insufficient function of either BLM or FANCD2 would show similar biological effects. To this end, we performed a complete set of proliferation assays via U2OS derivative cells, isogenic to the status of FANCD2 activation or to the normal functional level of BLM. As shown in Figure 4A–4D, Supplementary Figure S3A and S3B, U2OS cells harboring a compromised FANCD2 activation or an insufficient BLM were found to be more sensitive to the treatment of UVB or MMC than their corresponding control cells. In addition, we decided to further confirm this observation in a different type of human cells. We used a set of similar stable cell pairs derived from ovarian cancer cell line PA-1 (Supplementary Figure S2A). Again, a similar cell proliferation/survival rate was displayed between PA-1 derivative cells either carrying a compromised FANCD2 activation or a malfunctioned BLM compared to their corresponding control cells (Supplementary Figure S3C–S3H). Together, these results validate a common platform shared by both BLM and FANCD2 to act in response to DNA damage.

### Naturally-occurring mutations at domain VI of BLM (deletion or point mutation) are associated with the high grade ovarian tumor

**Figure 5:**
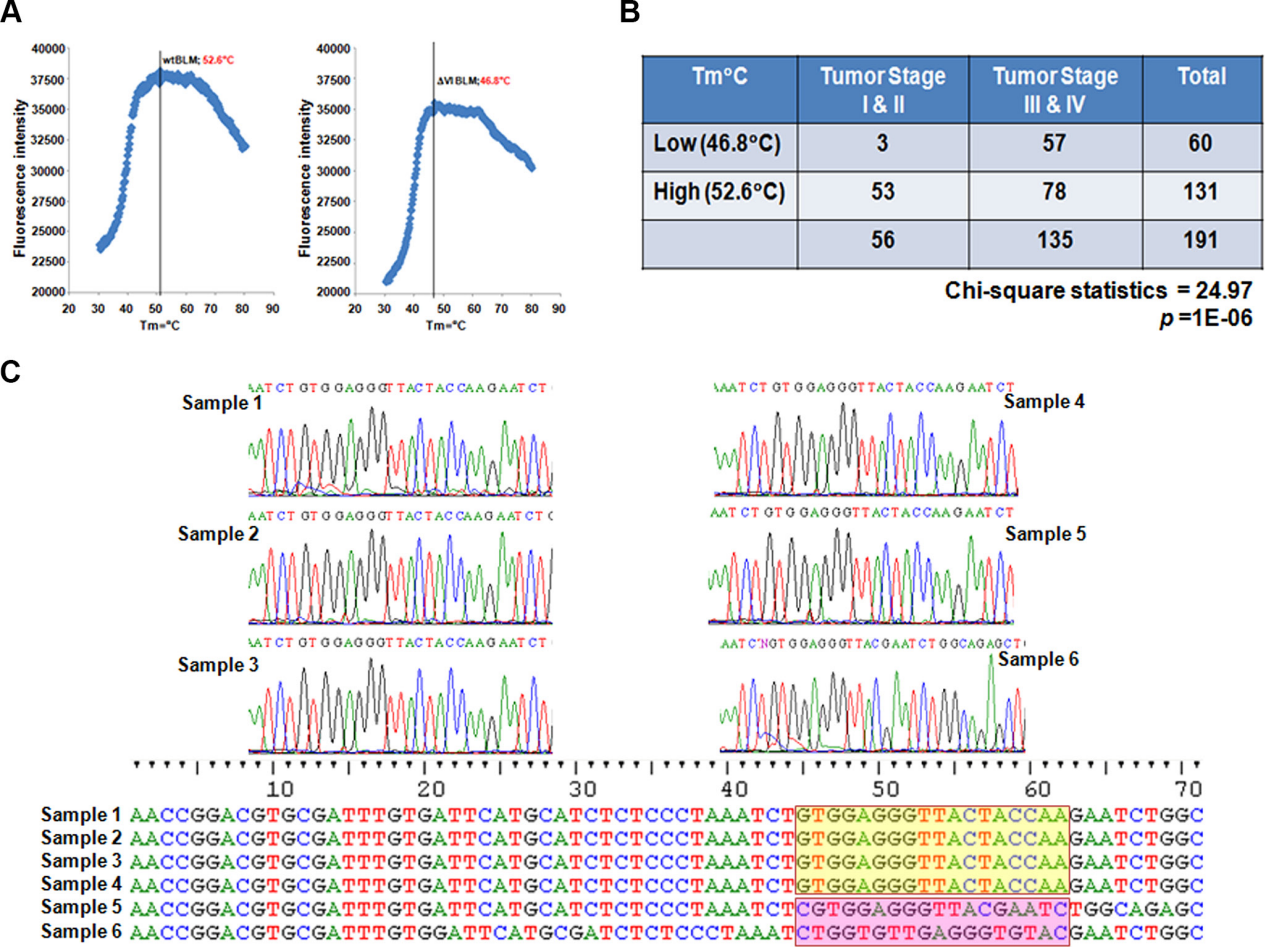
Functional Importance of BLM Regulation of FANCD2 Activation (**A**) Establishment of the modified TaqMan Assay. FAM-probe detecting PCR products of wtBLM cDNA-template shows a florescent peak at a Tm = 52.6°C; but for mtBLM CDNA at motif IV shows a florescent peak at a Tm = 46.8°C. (**B**) Summarization of 191 samples' TaqMan results. Samples having a low Tm (around 47°C) appear to be statistically associated with tumors at high stages (III or IV). A Chi-square test was performed with a *p* value = 1E-06, statistics 24.97 at *p* < 0.05 level. (**C**) Sequence verification of the modified TaqMan Assay. 3 samples were randomly picked up from low or high-Tm groups, respectively (a total of 6 samples were used for sequencing). The sequence data confirmed the low-Tm samples indeed carry mutations in the tested region (red shad). Whereas the high-Tm samples do have the sequences matched well with the wild type one (Yellow shad) (Noting that one sample from low-Tm group also carries a wild type sequence, 2/3 samples as expected).

To explore an immediate opportunity for prevention and diagnosis of DNA damage-related diseases including UV-driven skin cancers, we modified the conventional TaqMan assay at the annealing stage to differentiate the peaks of probe fluorescence bound to wt and mt templates (Blm cDNA) respectively (Figure 5A) (the probe sequence was designed upon the Y974Q975 region of BLM: FAM-GGTTACTACCAAGAATCT). As anticipated, wtBlm cDNA showed a peak annealing temperature of 52.6°C; while ΔY974Q975 Blm cDNA optimally bound to the probe at 46.8°C. This probe was subsequently used to detect mutations occurring at the Y974Q975 region of BLM in human cancer. We used this modified TaqMan method to examine 191 human ovarian cancer cDNA samples with a clear documentation of tumor stage and grade [34–36]. We found that the TaqMan probe could anneal to the PCR products of ~60 samples with a Tm around 47°C, and the rest of the samples with a Tm around 53°C. Among the low-Tm-carrying samples, the majority were derived from high-stage tumors; whereas in high-Tm-carrying samples, only half were derived from the high-stage tumors. When Chi-square is applied, high or low-Tm is reversely associated with tumor stages with a *p* value of 1E-06 (Figure 5B). We also randomly picked up several sample PCR-products for sequencing, and found that the low-Tm carrying samples indeed harbor mutations in the region examined, and that the high-Tm-carrying samples matched well with the wt sequence (Figure 5C). This is a very exciting finding, because these results may lead to a potential in predicting ovarian cancer grade/stage at the genetic level. In addition, UV damage is the primary cause of skin cancer. Clinically, skin cancer patients are seen primarily in the setting of a dermatologist's office lacking sequencing equipment for testing. Therefore, there is a large unmet need for a rapid diagnosis of pre-lesions of UV-driven cancer. As such, PCR-based tests would be considerably more cost effective compared to genome sequencing. Importantly, this finding further shows the functional importance of the BLM domain VI in human tumorigenesis, and that compromised FA signaling can be a strong contributor to the tomorigenicity originating from the mutated Blm gene.

## DISCUSSION

The discovery that the BLM and FA proteins share the same protein complex [37] indicates an important connection for their roles in genome maintenance. Recent work investigating the relationship between BLM and FA proteins, such as FANCD2, shows that the function of BLM in DNA replication is dependent upon FANCD2 protein that is seemingly independent of its role in the canonical FA signaling pathway [38–40]. Here, we report that BLM is involved in the early activation/monoubiquitination of FANCD2 in response to the DNA crosslinking agents: Cisplatin, MMC, or UV. In BLM null cells in comparison to BLM sufficient cells, FANCD2 activation was delayed following the treatment of UV, Cisplatin or MMC, all of which lead to DNA crosslinking damage. This suggests that the level of BLM expression plays a critical role for the timely activation of FANCD2. However, in the artificial Blm “null” U2OS cells via a RNA silencing approach, the U2OS cells treated with UV showed a similar delay to the observations in BS cells, this was not observed in the cells treated with Cisplatin or MMC. Interestingly, the duration of FANCD2 activation is shorter in Blm compromised cells compared to their control cells with a normal level of BLM protein expression. This discrepancy may result from the severity of DNA damage that demands a full level of BLM or a certain level of BLM. As such, the degree of DNA damage triggered by UV might be more severe than that initiated by Cisplatin or MMC. We believe that different patterns of DNA crosslink damages (intra or inter-strand DNA crosslinks) may act differently in a cell context dependent manner. Nonetheless, the crosslinked DNA can activate the FA signaling pathway and mechanisms behind might be different upon a different type of crosslinks, but need to be further investigated.

Recently, FANCM was found to be a common link between BLM and FA signaling [41]. Given that FANCM is an essential member of the complex E3 accounting for the monoubiquitination of FANCD2, we investigated whether FANCM expression was dependent upon BLM. This was done by using cells carrying a sufficient or deficient level of BLM expression, which would help our understanding of the reduced magnitude in FANCD2 monoubiquitination. However, the level of FANCM expression in both types of cells remained constant (Supplementary Figure S4). Therefore, it awaits the further investigations into the relationship between BLM and FANCD2 activation is required.

The motif VI of BLM together with those of I, II, III, IV and VI are generally known as the binding pocket for ATP binding and hydrolysis [31]. Although, its exact role in BLM functions is rarely discussed. Our study is the first to show that the motif VI of BLM can contribute to the regulatory role of BLM in the early activation of FANCD2. More importantly, we provide studies to demonstrate its functional significance. As such, naturally occurring mutations in the motif VI of BLM can interfere with the early activation of FANCD2. BLM has been shown to actively work with other FA proteins, such as FANCJ [42] and FANCM as mentioned before. In addition, BLM function appears to be inhibited by non-ubiquitinated FANCD2 in DNA replication [38, 39]. Our study on the dependency of timely activation of FANCD2 on BLM adds an additional layer of complexity into the relationship between BLM and FA proteins. Clearly, more studies are required to continue elucidating how these cancer susceptibility gene products work in concert to govern genome stability and thus, enhancing our understanding of genomic instability, as seen in aging and cancer.

## MATERIALS AND METHODS

### Cell lines, antibodies, and chemicals

PSNF5 and PSNG13 are two BS derivative cell lines isogeneic to BLM expression. PSNF5 is a complemented BS cell line with wild-type BLM cDNA from PSNG13 BS line [30, 43]. U2OS and PA-1 cells were purchased from American Type Culture Collection (ATCC; Manassas, VA) and maintained in DMEM medium supplemented with 10% fetal bovine serum at 37°C in 5% CO^2^ (v/v). Antibodies against the FANCD2 and BLM were obtained from NOVUS. The anti-Flag and β-actin antibodies as well as the chemicals Mitomycin C (MMC), Cisplatin, crystal violet solution and Puromycin were purchased from Sigma.

### Gene knockdown by lentivirus-mediated shRNA targeting Blm or FANCL

For lentiviral transduction essentially as described previously [44]: a set of pLKO.1 plasmids containing shRNA targeting BLM (Forward Primer; 5′-CCGGAGAGTCAATTCAG AATTATCTCGAGATAA TTCTGAATTGACTCTTTTT TG-3′ Reverse Primer; 5′ -A ATTCA AAAAAGAGTCAATTCAGAATTATCTCG AGATAATTCTGAATTGACTCT-3′), along with pLKO.1 empty vector were used to generate corresponding lentivirus. A set of five pLKO.1 plasmids containing shRNA targeting FANCL (purchased from Thermo scientific, Open BioSystems) along with the pLKO.1 empty vector was used to generate corresponding U2OS and PA-1 cells with silenced FANCL. Infected cells were pool-selected with Puromycin for one week, and BLM or FANCL knockdown was verified using BLM and FANCL antibodies, respectively (NOVUS).

### Plasmid construction

As described previously [45, 46]: we created various versions of Blm cDNA plasmids encoding Blm proteins with a deleted motif of I, Ia, II, III, IV, V, or VI. Primers used to generate those mutants are shown in Table below.

**Table:** 

BLM primer	5′-3′ DNA sequences
Bloom Motif I+Ia	F; gcg atc aat gct gca ctg ctt caa aag ctg act tcc ttg gat
	R; atc caa gga agt cag ctt ttg aag cag tgc agc att gat cgc
BLM Motif II	F; cta ctc tgg aga atc tct ata gtc agt ggg gac atg at
	R; atc atg tcc cca ctg act ata gag att ctc cag agt ag
BLM Motif III	F; ttt cct tct gtt ccg gtg ctg act cag ctg aag att c
	R; gaa tct tca gct gag tca gca ccg gaa cag aag gaa a
Bloom Motif IV	F; tgt gat tca tgc atc tct cga aat atc tca ctg cct gct t
	R; aag cag gca gtg aga tat ttc gag aga tgc atg aat cac a
Bloom Motif V	F; att aat cag gat ggc tgt att cat gca tct ctc cct
	R; agg gag aga tgc atg aat aca gcc atc ctg att aat
Bloom Motif V+VI	F; tgg att aat cag gat ggc tgt gaa ata tct cac tgc ctg ctt
	R; aag cag gca gtg aga tat ttc aca gcc atc ctg att aat cca
Bloom Motif VI	F; ccg gac gtg cga ttt gtg gaa ata tct cac tgc ctg
	R; cag gca gtg aga tat ttc cac aaa tcg cac gtc cgg

### Immunofluorescence staining

The Immunofluorescence staining was performed as previously described [47]. PSNF5, PSNG13, U2OS and PA-1 stable cells (1 × 10^3^) were seeded onto sterilized black well slides and fixed with pre-chilled 3.7% paraformaldehyde/PBS for 15 min at 4°C and quenching with 125 mM of glycine for 5–10 min at room temperature (RT). Next, these cells were permeabilized with 0.2% pre-chilled Triton-X 100/PBS for 5 min at 4°C, blocked with 3% horse serum/PBS for 30 min at RT, and subsequently incubated with FANCD2 antibodies at 4°C overnight. After washing, cells were further incubated with anti-rabbit secondary antibodies at 1:1000 dilutions for 30 min at RT. After the incubation with secondary antibodies, cells were washed and mounted in Vectashield mounting media with DAPI (Vector Laboratories, Burlingame, CA). Cells were observed with a 40X objective lens under an Olympus BX-51 microscope equipped with a SenSys fluorescence CCD camera. Images were acquired and analyzed using MetaMorph version 4.5 Premier.

### Cell survival assay

Equal numbers of U2OS and PA-1 stable cells were seeded into 35 mm dishes one day prior to MMC treatment. Cells were treated with MMC at concentrations of 50 ng/ml, 25 ng/ml, 12.5 ng/ml, or 6.25 ng/ml MMC for 5 days. Resulting colonies were then fixed and stained with crystal violet solution. The numbers of colonies were counted (Each drug dose was tested in triplicate) using a GelDoc with Quantity One software (BioRad).

### Modified TaqMan assay

We designed a set of primers 50bp up and down the region encoding Y974Q975 of BLM and FAM-probe (18 mers) covering right on the region. Oligos (Forward Primer; TGATTCATGCATCTCTCCCTAAA, Reverse Primer; CTGGTCACATCATGATAGGTATAGAA, Probe; GGTTACTACCAAGAATCT and Sequence Primer; CAGGATGGCTGTCAGGTTATC) were ordered from IDT. 1 pg of wtBLM or ΔY974Q975-Blm cDNA was used as the template for the controls to set up the assay condition. The TaqMan assay was performed using 0.5°C increments for 5 seconds from 30 to 70°C until the final annealing stage. The intensity of FAM-florescence was plotted in referring to the increment of Tm. The peak of florescence was clearly separated using our modified program at 52.6°C for wtBLM cDNA and 46.8°C for Y974Q975-Blm cDNA.

### Tissue sample TaqMan assay

Frozen dissected ovarian tumor specimens, examined by two independent pathologists, contained 80–90% tumor cells, were pulverized manually in liquid nitrogen for extraction of total RNA following a standard phenol–chloroform protocol. The RNA samples were treated with DNase to remove genomic DNA contamination and were concentrated using the RNeasy MinElute Cleanup kit (Qiagen Inc., Valencia, CA). The quality of ribonuclease-free deoxyribonuclease-treated total RNA was determined using 1% agarose gel electrophoresis for the integrity of 18s and 28s ribosomal RNAs. 1 μg of total RNA from each sample was processed for conversion to cDNA using the Cloned AMV First-Strand cDNA Synthesis kit (Invitrogen, Carlsbad, CA). cDNA samples from 191 patients were used for the TaqMan assay. Primers (Forward Primer; TGATTCATGCATCTCTCCCTAAA, Reverse Primer; CTGGTCACATCATGATAGGTATAGAA, Probe; GGTTACTACCAAGAATCT and Sequence Primer; CAGGATGGCTGTCAGGTTATC) were designed in-house and chemically synthesized by Integrated DNA Technologies (IDT, Coralville, IA). The PCR conditions were modified as described above for our modified TaqMan assay. The calculation of BLM products from the real-time PCR was independently performed, and performers were blinded to the clinical data.

### Statistical analysis

The correlation between the Tm's and the cancer stage was analyzed using the Chi-square test. A *p*-value < 0.05 was considered statistical significance. All analyses were processed by SPSS 20 software.

## SUPPLEMENTARY FIGURES


