# Supplementary material for: BLM promotes the activation of Fanconi Anemia signaling pathway
Source: Oncotarget. 2016 Apr 12;7(22):32351–61. doi: 10.18632/oncotarget.8707 (PMC5078018; doi:10.18632/oncotarget.8707)
Supplement: Supplementary file 1 [file oncotarget-07-32351-s001.pdf]

BLM promotes the activation of fanconi anemia signaling pathway

Supplementary Materials

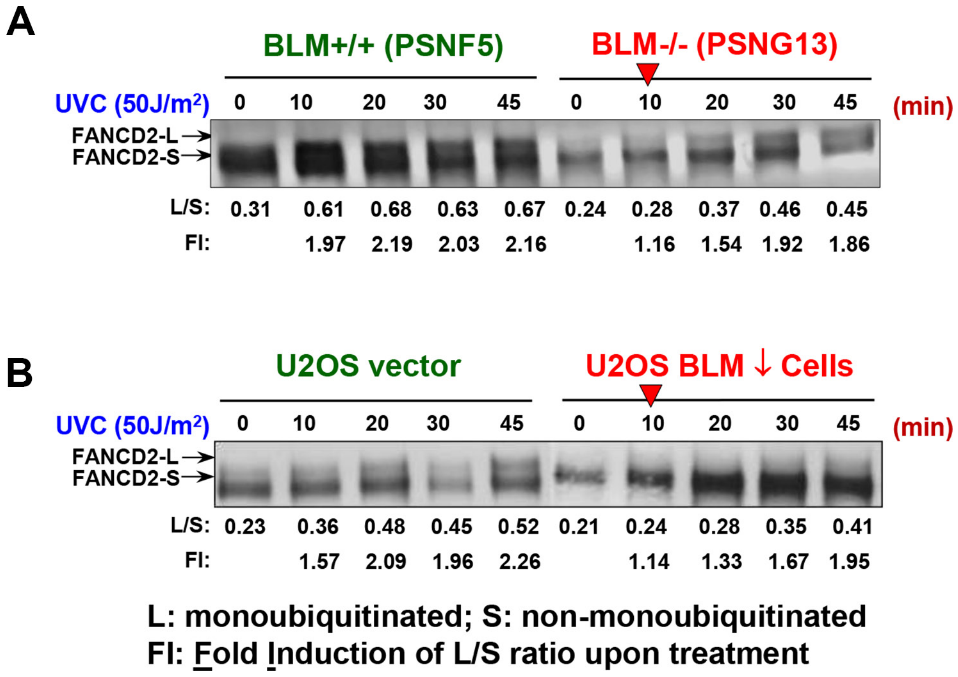

Supplementary Figure S1: The magnitude of FANCD2 activation is reduced in BS cells (A) or BLM-silenced U2OS cells treated with UVC (B). Red arrowheads indicate the delayed activation of FANCD2. The band density was determined by Image J software, and the ratio of FANCD2-L (monoubiquitinated FANCD2 isoform) to FANCD2-S (un-monoubiquitinated form) was calculated (L/S). Fold induction (FI) is the L/S ratio of the treated over the untreated.

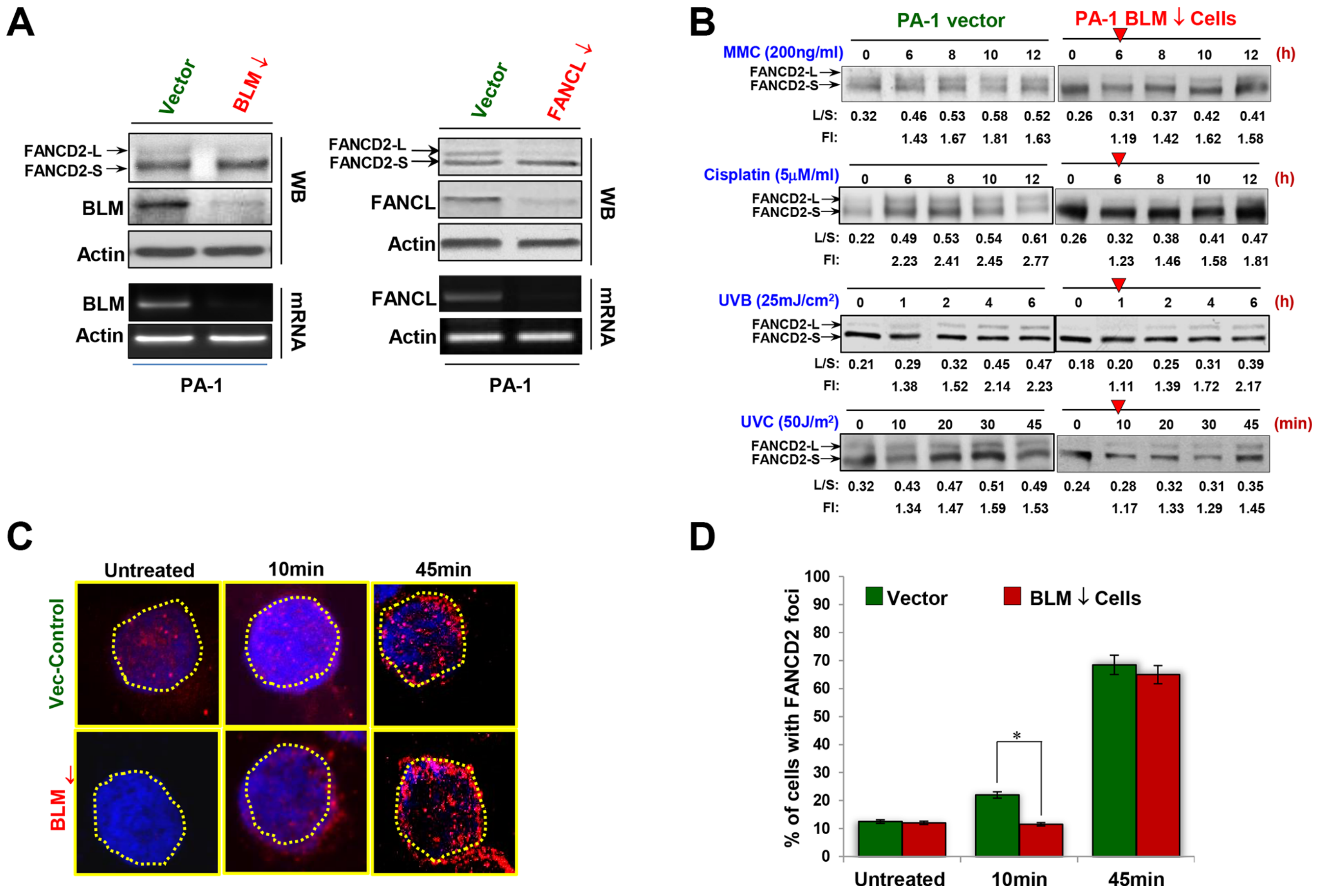

Supplementary Figure S2: (**A**) PA-1 cells carrying either shRNA targeting Blm or empty vector were established. The levels of both BLM mRNA and protein were downregulated compared to control cells. The corresponding level of monoubiquitinated FANCD2 is compromised in BLM-downregulated cells. PA-1 stable cells isogenic to the basal level of monoubiquitinated FANCD2 expression were established via silencing FANCL. (**B**) FANCD2 activation is compromised in BLM-deficient PA-1 cells when treated with MMC, Cisplatin, UVB and UVC. As red arrowheads indicated, BLM-downregulated cells showed a delayed activation of FANCD2 compared to their corresponding control cells. The levels of S and L isoforms of FANCD2 are expressed as the L:S ratio. Fold induction (FI) is the L:S ratio of treated: untreated. (**C**) FANCD2 foci are decreased in BLM-silenced PA-1 cells. The same batch of PA-1 stable cells, used for Western blotting shown in Supplementary Figure S2B, was also prepared for the immunofluorescent study. Cells were treated with UV (50 J/m^2) as described in the Materials and Methods. Again, BLM-downregulated PA-1 cells carry a lower density of FANCD foci compared to their control cells (Foci represent the monoubiquitinated/ activated FANCD2). (**D**) Quantification of FANCD2 focus formation. The number of cells carrying FANCD2 foci was measured in 120 cells for each time point. The graph shows the mean and SD of the percentage of cells with > 10 FANCD2 foci. Statistical significance was calculated using the Pearson's chi-square test. Data are the means of 3 independent determinations.

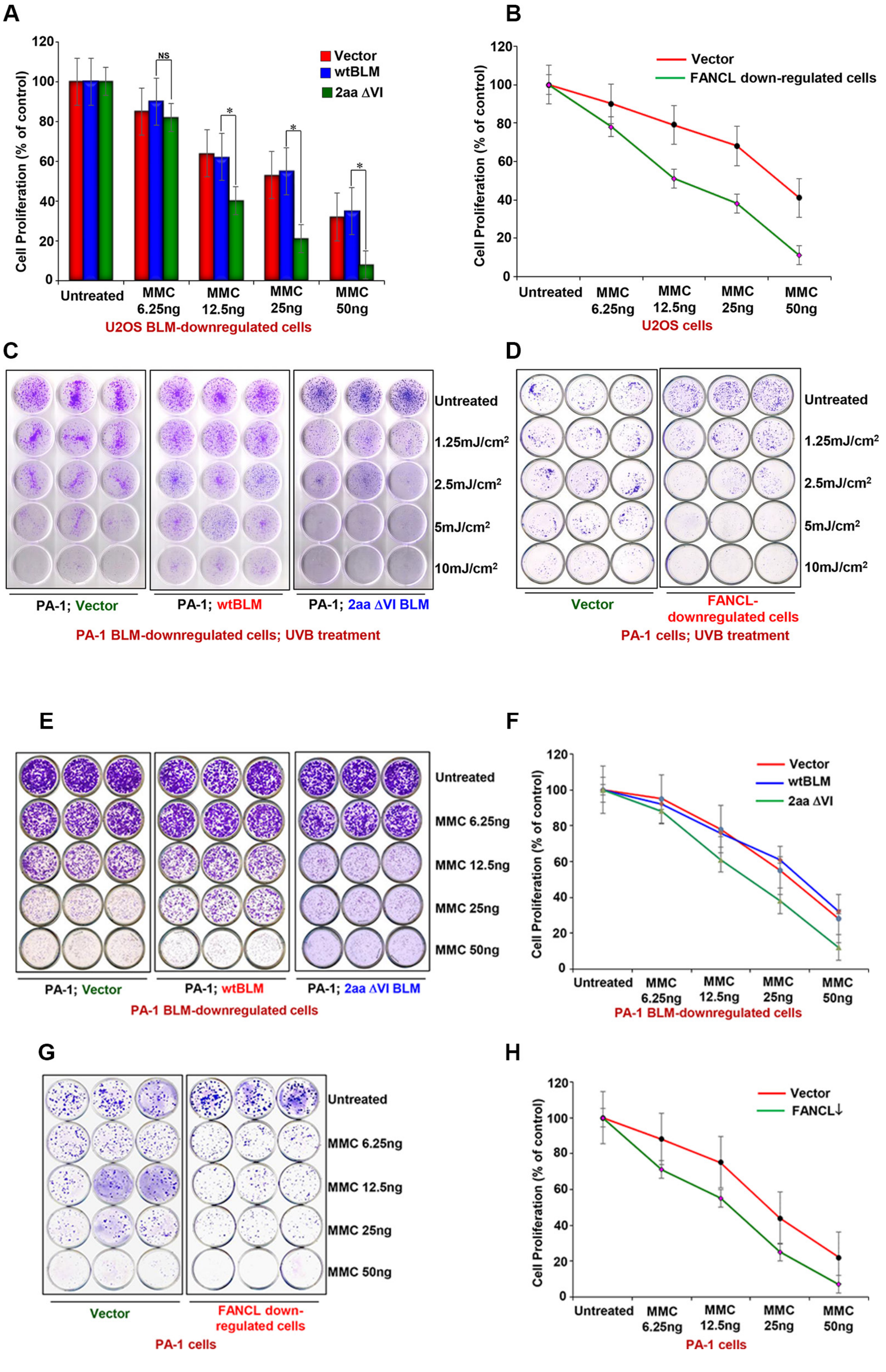

Supplementary Figure S3: (A) BLM-downregulated or (B) FANCL-downregulated U2OS cells were more sensitive to MMC treatment than their corresponding control cells. BLM-downregulated (**C**) or FANCL-downregulated (**D**) PA-1 cells were more sensitive to UVB treatment than their corresponding control cells. This is similar to what is seen in these cells treated with MMC (**E**) and (**G**). Representative results of at least three independent experiments assessed by crystal violet staining are shown and the survival curves plotted upon the quantification of cell proliferation are shown in (**F**) and (**H**) respectively (Means ± SD of 3 independent experiments). The asterisks denote *$p < 0.05$.

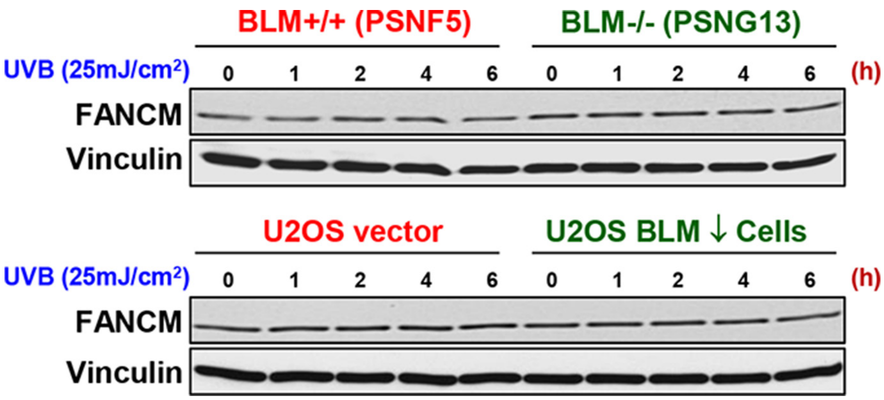

Supplementary Figure S4: Expression levels of FANCM protein in BS cells and BLM-silenced U2OS cells treated with UVB. There are no alterations in FANCM protein levels in both types of cells carrying a deficient Blm or compromised FA signaling when compared to corresponding controls harboring a sufficient Blm or intact FA signaling, respectively.
